# Tetrandrine Induces Apoptosis of Human Nasopharyngeal Carcinoma NPC-TW 076 Cells through Reactive Oxygen Species Accompanied by an Endoplasmic Reticulum Stress Signaling Pathway

**DOI:** 10.3390/molecules21101353

**Published:** 2016-10-12

**Authors:** Ya-Jing Lin, Shu-Fen Peng, Meng-Liang Lin, Chao-Lin Kuo, Kung-Wen Lu, Ching-Lung Liao, Yi-Shih Ma, Fu-Shin Chueh, Kuo-Ching Liu, Fu-Shun Yu, Jing-Gung Chung

**Affiliations:** 1 Department of Biological Science and Technology, China Medical University, Taichung 40402, Taiwan; cathy263938@gmail.com (Y.-J.L.); sfpeng@mail.cmu.edu.tw (S.-F.P.); 2 Department of Medical Laboratory Science and Biotechnology, China Medical University, Taichung 40402, Taiwan; mllinsally@yahoo.com.tw (M.-L.L.); kchliu@mail.cmu.edu.tw (K.C.L.); 3 Department of Chinese Pharmaceutical Sciences and Chinese Medicine Resources, China Medical University, Taichung 40402, Taiwan; clkuo@mail.cmu.edu.tw; 4 College of Chinese Medicine, School of Post-Baccalaureate Chinese Medicine, China Medical University, Taichung 40402, Taiwan; prorna@mail.cmu.edu.tw (K.-W.L.); qbking@ms29.hinet.net (C.-L.L.); 5 Department of Chinese Medicine, E-Da Hospital, Kaohsiung 82445, Taiwan; m2367591@ms25.hinet.net; 6 School of Chinese Medicine for Post-Baccalaureate, I-Shou University, Kaohsiung 82445, Taiwan; 7 Department of Health and Nutrition Biotechnology, Asia University, Wufeng, Taichung 41354, Taiwan; fushin@asia.edu.tw; 8 School of Dentistry, China Medical University, Taichung 40402, Taiwan; 9 Department of Biotechnology, Asia University, Wufeng, Taichung 41354, Taiwan

**Keywords:** tetrandrine (TET), endoplasmic reticulum (ER) stress, reactive oxygen species (ROS), apoptosis, NPC-TW 076 cells

## Abstract

Nasopharyngeal carcinoma (NPC) is an epithelial malignancy of the head and neck and the incidence is higher in Southeast Asia. Tetrandrine (TET) is a bisbenzylisoquinoline alkaloid, a natural product, and exhibits biological activities including action against many human cancer cell lines. However, the molecular mechanism of TET-induced cell apoptosis in human NPC cells is still unclear. In the present study, we investigated TET-induced apoptotic cell death and associated possible signal pathways on human nasopharyngeal carcinoma NPC-TW 076 cells in vitro. Phase contrast microscopy was used to examine cell morphology and DAPI staining was used to examine chromatin condensation. Flow cytometry assay was used to measure total viable cells, cell cycle and sub-G_1_ phase distribution, reactive oxygen species (ROS), Ca^2+^, and mitochondria membrane potential (Δ*Ψm*) in NPC-TW 076 cells. Results indicate that TET induced cell death through the cell morphological changes, caused G_0_/G_1_ phase arrest, increased ROS and Ca^2+^ production, and finally caused apoptotic cell death in NPC-TW 076 cells. There was no influence on the level of Δ*Ψm* after TET treatment. Western blotting indicated that TET increased endoplasmic reticulum (ER) stress associated protein expression such as GADD153, GRP78, ATF-6α and ATF-6 βwhich indicated that TET induced cell death through ER stress. ER stress is a potential target in cancer treatment, so the ability of TET to induce ER stress response and to activate programming cell death in NPC-TW 076 cells make this molecule become a promising anticancer agent.

## 1. Introduction

Nasopharyngeal carcinoma (NPC) is an endemic disease particularly in Southeast Asia and Southern China [1,2]. In Taiwan, NPC is the fifteenth most common cancer and the mortality rate was 2.2 people per 100,000 population per year from NPC based on the 2016 report from the Department of Health [3]. Epidemic studies have shown that genetic, environmental and viral factors are associated with NPC development [4,5,6]. NPC is known to be sensitive to radiotherapy, thus, early non-metastatic patients with NPC used radiotherapy for treatment [7]. Currently, the treatment of NPC usually involves the combined use of radiotherapy and systemic chemotherapy [8], however, it still has side effects. Abundant evidences have demonstrated that new compounds from natural products, including Chinese herbal medicines, can reduce the side effects while treating NPC patients. Induced cancer cell apoptosis is recognized to be one of the best strategies for anticancer drug development.

Apoptosis, a programmed cell death, can be triggered by extrinsic (death receptor) pathway or intrinsic (mitochondrial dependent) pathway, or both [9]. The extrinsic pathway involves Fas (one of the tumor necrosis factor family of apoptosis induction receptors) and Fas-L followed by recruiting and activating procaspase-8 [10] and activated caspase-8 initiates downstream caspase-3 and triggers cell apoptosis. The intrinsic pathway involves the alteration of mitochondrial membrane potentials, followed by the release of cytochrome c from mitochondria into the cytosol and activates caspase-9 and cspase-3 for cell apoptosis. Thus, the mitochondria control mechanisms underlying apoptosis lead to the release of protein effectors such as cytochrome c, AIF and Endo G [11,12,13,14]. 

Tetrandrine (TET) is a bisbenzylisoquinoline alkaloid, a natural product, and is purified from the root of *Stephania tetrandra* (Hang fang ji) of the Menispermaceae and it has been shown to exhibit numerous biological activities such as antihypertensive and antiarrhythmic functions [15], immunomodulation [16], anticancer effects against several cancers [17,18,19,20], and increased animal survival time and survival rate in vivo [21,22,23,24]. Furthermore, in human drug-resistant esophageal squamous carcinoma cells, TET enhances the cytotoxicity of cisplatin via inhibition of multidrug resistance-associated protein 1 [25]. TET suppresses cancer angiogenesis and metastasis in 4T1 breast tumor-bearing BALB/c mice [26]. TET exhibited strong inhibitory effect on human prostate cancer cell proliferation, migration, and invasion in vitro [27]. However, TET revealed a potential therapeutic effect on nasopharyngeal cancer and was able to sensitize the human nasopharyngeal carcinoma CNE cells under radiation therapy [28].

Anti-cancer effects of TET have been reported in various cancer cell lines in vitro or in vivo. However, few reports have described about the anti-cancer effect of TET on human nasopharyngeal carcinoma cells. In this study, we investigated the effects of TET and the molecular mechanism of TET on the induction of apoptosis in human nasopharyngeal carcinoma NPC-TW 076 cells. Our results suggest that TET-induced cell apoptosis through endoplasmic reticulum stress signaling pathway in human nasopharyngeal carcinoma NPC-TW 076 cells.

## 2. Results

### 2.1. TET Induced Cell Morphological Changes and Decreased the Total Viable Cell Number in NPC-TW 076 Cells

**Figure 1:**
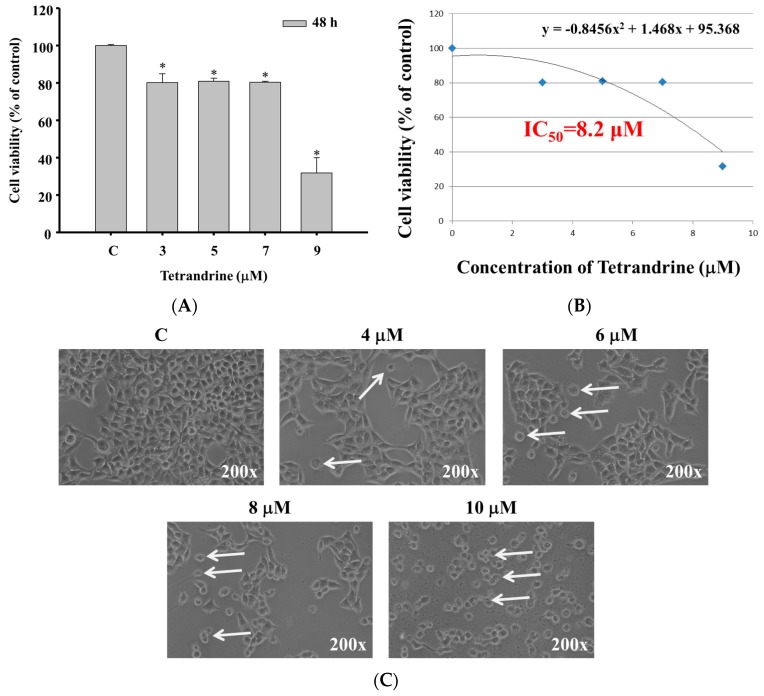
TET decreases the number of viable NPC-TW 076 cells and induced cell morphological changes in vitro. Cells were treated with TET at a concentration range of 0–10 μM for 48 h and then the cells were collected for the percentage of viable cell measurements (**A**) by flow cytometry as described in Materials and Methods. IC_50_ is examined to be 8.2 μM (**B**). Cells were examined and photographed for cell morphological changes by contrast-phase microscopy at 200× (**C**) or * *p* < 0.05, significant difference between TET-treated groups and the control as analyzed by Student’s t test.

The NPC-TW 076 cells were treated with different concentrations of TET for 48 h. As shown in Figure 1A,B, TET treatment significantly reduced total viable cell number (Figure 1A) at 48 h treatment with an IC_50_ of 8.2 μM (Figure 1B). TET treatment (4–10 μM) obviously induced cell morphological changes compared to the control (Figure 1C).

### 2.2. TET Induced Nuclear Condensation in NPC-TW 076 Cells

**Figure 2:**
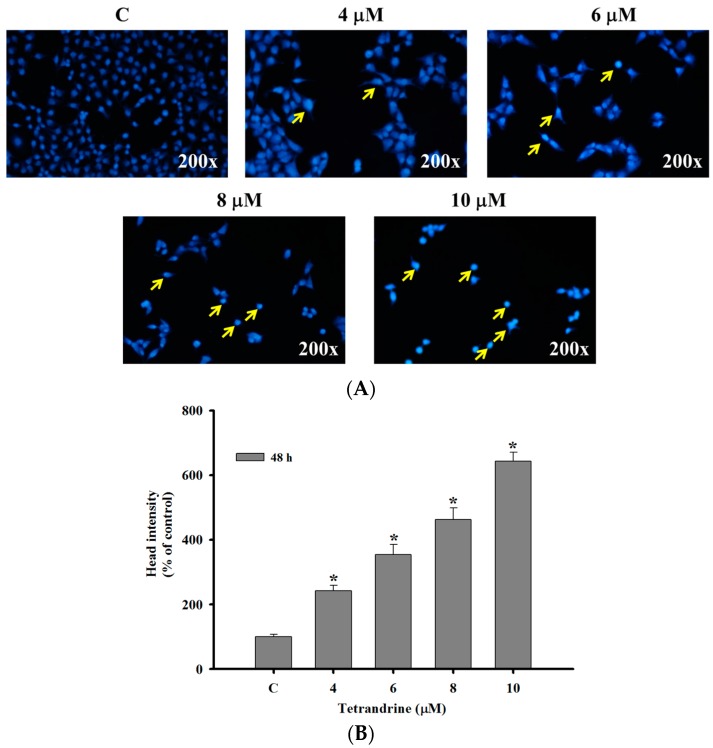
TET induces nuclear chromatin condensation in NPC-TW 076 cells. Cells were treated with 0, 4, 6, 8 and 10 μM of TET for 48 h and then were stained with DAPI as described in Materials and Methods. Cells were examined and photographed using a fluorescence microscope at 200× (**A**) and the DAPI fluorescence intensity were quantified (**B**). * *p* < 0.05, significant difference between TET-treated groups and the control as analyzed by Student’s t test.

NPC-TW 076 cells were treated with TET (0–10 μM) for 48 h and then were stained with DAPI, photographed by fluorescence microscopy and the results are shown in Figure 2. Figure 2A,B indicated that higher TET concentration led to brighter DAPI fluorescence of NPC-TW 076 cells after 48 h treatment when compared to control. Furthermore, the higher TET concentration results in lower cancer cell number (Figure 2A). The bright fluorescence means that cells have nicked DNA and nuclear chromatin condensation.

### 2.3. TET Induced G_0_/G_1_ Phase Arrest and Sub-G_1_ Phase in NPC-TW 076 Cells

**Figure 3:**
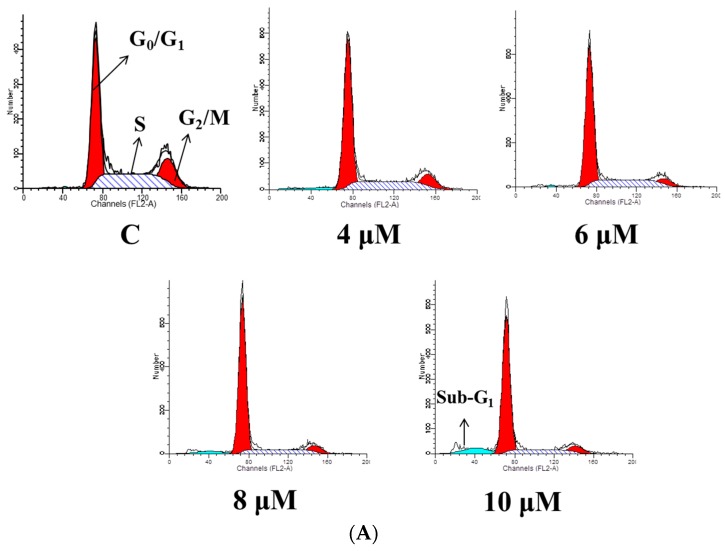

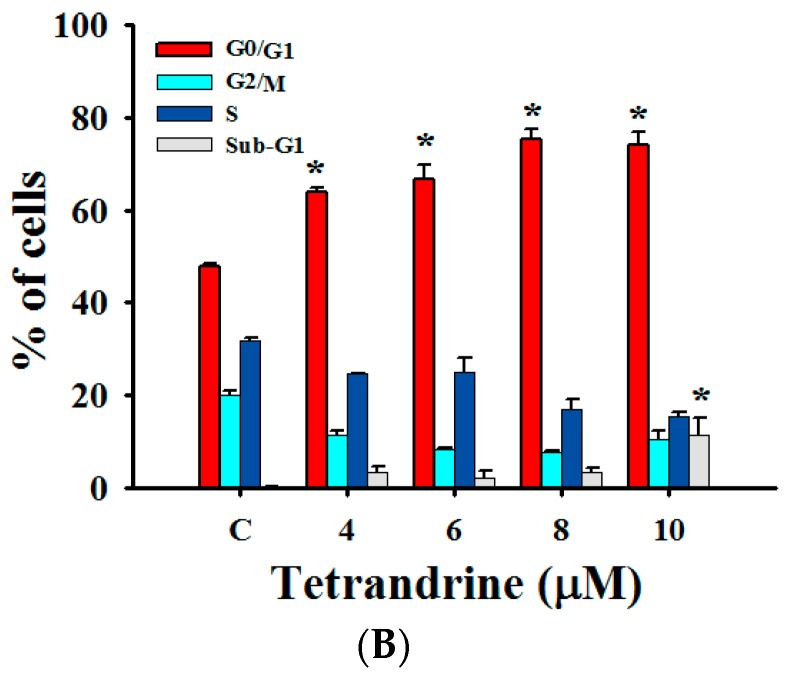
TET induces G_0_/G_1_ phase arrest and sub-G_1_ phase in NPC-TW 076 cells. Cells (1 × 10^5^ cells/well) in 12-well plates and were incubated with TET (0–10 μM) for 48 h. Cells were harvested and fixed in 70% ethanol and then incubated with a solution containing 50 mg/mL PI and 50 μg/mL RNase A for 30 min in the dark at 37 °C. Cells were analyzed for cell cycle distribution (**A**) by flow cytometer or were evaluated for the percentage of cell cycle distribution (**B**) as described in Materials and Methods.

In order to understand whether TET decreased cell number via cell cycle arrest and/or induced apoptotic cell death, NPC-TW 076 cells were treated with 0, 4, 6, 8 and 10 μM of TET for 48 h. Cells were collected to analyze cell cycle distribution and sub-G_1_ phase and the results are shown in Figure 3. The results indicated that TET induced G_0_/G_1_ phase arrest (Figure 3A) and these effects are dose-dependent (Figure 3B). Results also show that TET induce sub-G_1_ phase (apoptosis) in NPC-TW 076 cells (Figure 3A,B).

### 2.4. TET Induced Reactive Oxygen Species (ROS) and Ca^2+^ Productions but no Change in the Levels of Mitochondrial Membrane Potential (ΔΨm) in NPC-TW 076 Cells

**Figure 4:**
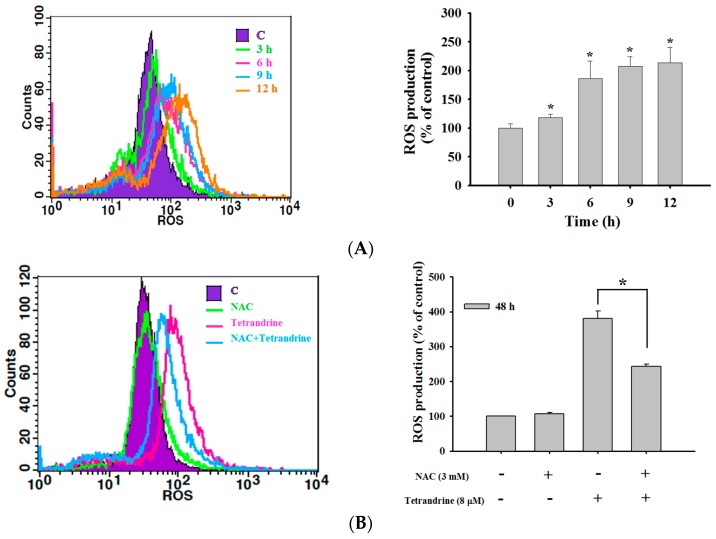

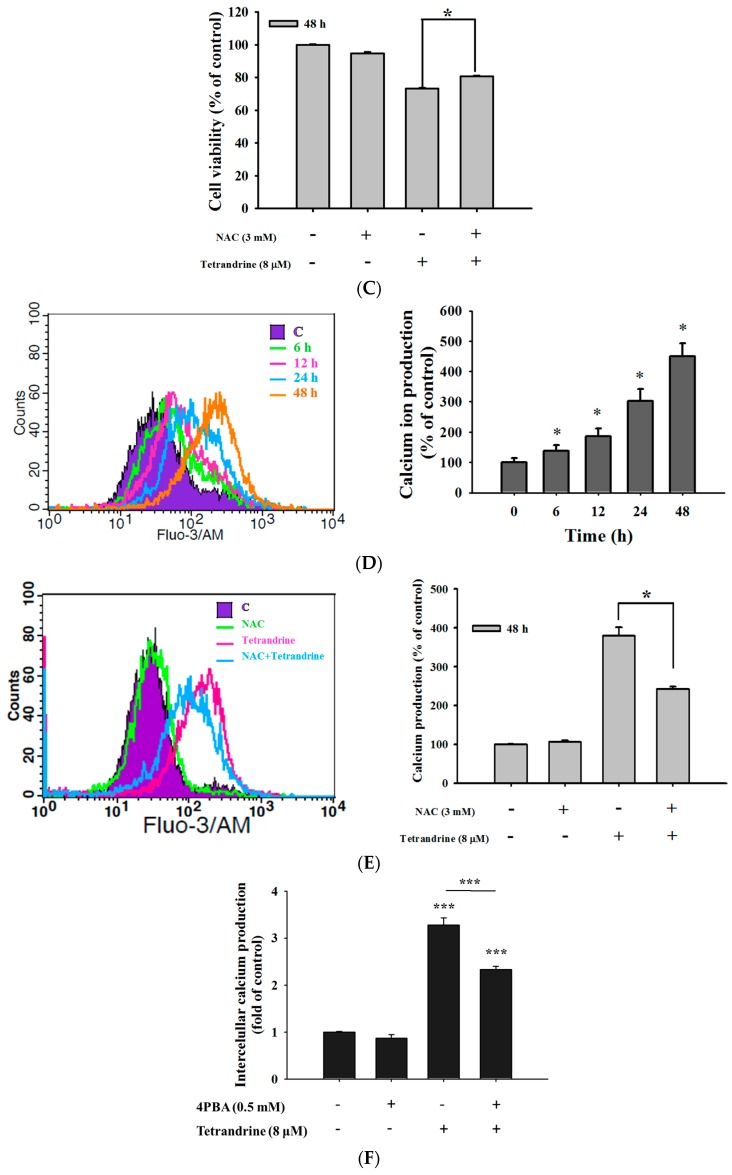

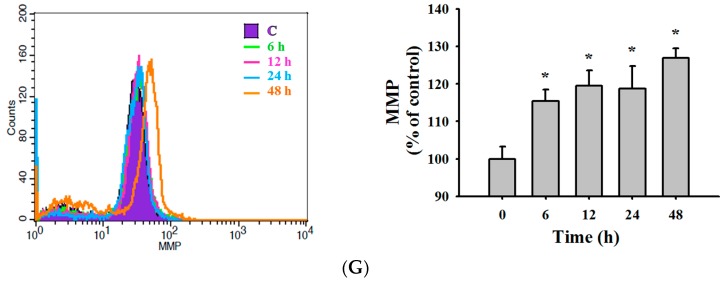
TET induces reactive oxygen species (ROS) and Ca^2+^ production and affects the levels of mitochondrial membrane potential (*ΔΨm*) in NPC-TW 076 cells. Cells (1 × 10^5^ cells/well) were treated with TET (8 μM) for various time periods. Cells were isolated and then were re-suspended in DCFH-DA for ROS (H_2_O_2_) (**A**) and cells were pretreated with NAC and then were measured for ROS (**B**) and the number of viable cells (**C**), re-suspended in Fluo-3/AM for further intracellular Ca^2+^ concentrations (**D**) and cells were pretreated with NAC (**E**) or 4PBA (**F**) and then were measured for Ca^2+^ production, and re-suspended in DiOC_6_ for the levels of *Ψm* measurement (**G**) as described in Materials and Methods. The results are shown as a mean ± SD (*n* = 3); * *p* < 0.05 or *** *p* < 0.001 significant difference between TET-treated groups and the control as analyzed by Student’s t test.

In order to investigate whether the production of ROS and Ca^2+^ or dysfunction of mitochondrial involved TET induced cell death in NPC-TW 076 cells, cells were treated with 8 μM of TET for various time periods and the results are shown in Figure 4. As shown in Figure 4A, TET increased ROS production during 6–12 h treatment. Cells pretreated with NAC (scavenger of ROS) and then treated with TET resulted in decreased ROS production (Figure 4B) but increased total viable cells when compared to TET treated only (Figure 4C). TET increased Ca^2+^ production during 6–48 h treatment (Figure 4D), however, NAC or 4PBA (ER stress blocker) pretreatment led to decreased Ca^2+^ production when compared to TET treatment only (Figure 4E). However, TET did not obviously affect the levels of mitochondrial membrane potential (*ΔΨm*) from 6 h up to 48 h treatment (Figure 4F). These results indicated that ROS and Ca^2+^ are involved in TET induced cell apoptosis in NPC-TW 076 cells in vitro.

### 2.5. TET Altered Apoptosis Associated Protein Expression in NPC-TW 076 Cells

**Figure 5:**
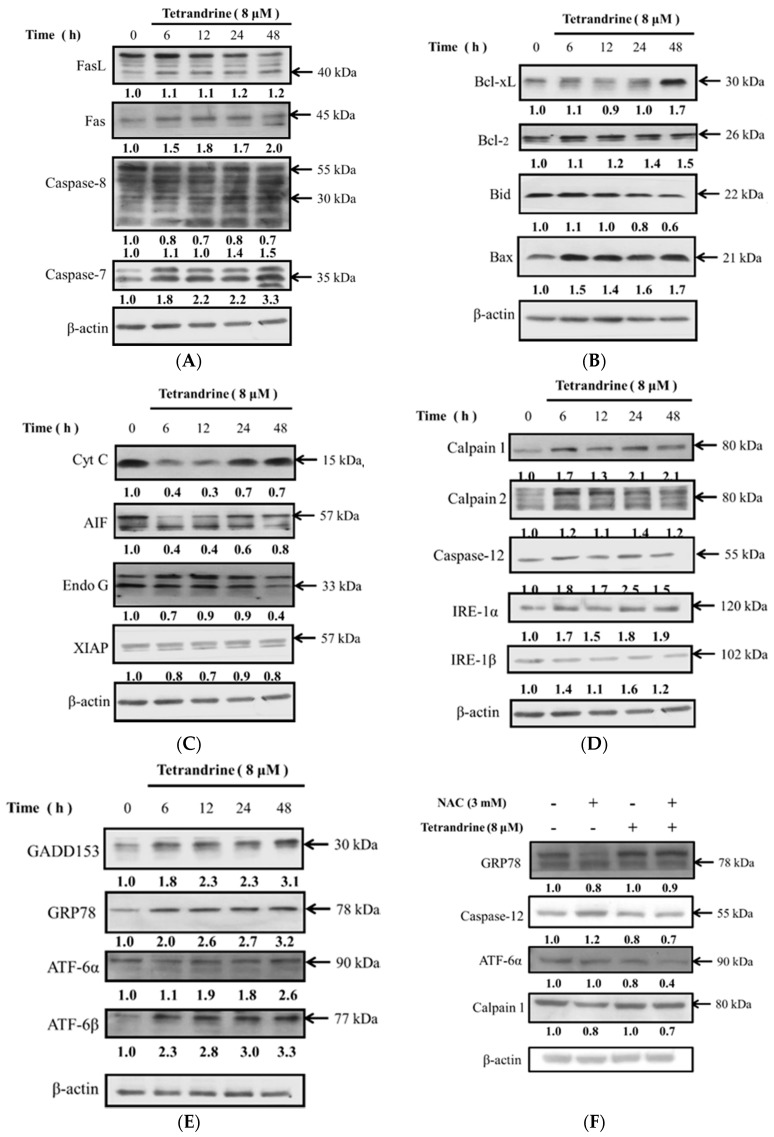

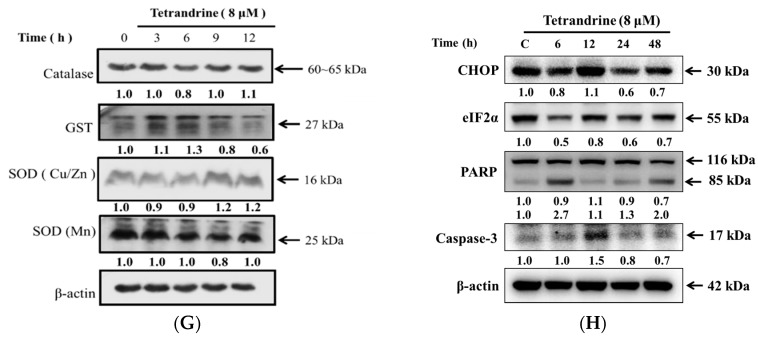
TET affects apoptosis associated protein expression in NPC-TW 076 cells. Cells were treated with 8 μM of TET for 0, 6, 12, 24 and 48 h and then total proteins were quantitated and apoptosis associated proteins were measured by western blotting as described in Materials and Methods. (**A**): FasL, Fas, caspase-8 and -7; (**B**): Bcl-xL, Bcl-2, Bid and Bax; (**C**): Cyto C, AIF, Endo G and XIAP; (**D**): Calpain 1 and 2, caspase-12, IRE-1α and IRE-1β; (**E**): GADD153, GRP78, ATF-6α and ATF-6β; (**F**): GRP78, caspase-12, ATF-6α and ATF-6β; (**G**): Catalase, GST, SOD (Cu/Zn) and SOD (Mn); (**H**): CHOP, eIF2α, PARP, and caspase-3.

Western blotting analysis was used to investigate the effects of apoptotic cell death associated protein expression induced by TET in NPC-TW 076 cells. NPC-TW 076 cells were treated with TET for various time periods and protein expressions were measured and results are shown in Figure 5. The results showed that TET significantly increased the expression of FasL, Fas, cleaved caspase-8, caspase-7 (Figure 5A), Bax, Bcl-xL, and Bcl-2 (Figure 5B), however, cyto c, AIF and Endo G (Figure 5C) were decreased. Based on these observations, TET induced cell apoptosis was not involved in mitochondria dysfunction. TET increased calpain 1, calpain 2, caspase-12, IRE-1α, IRE-1β (Figure 5D), GADD153, GRP78, ATF-6α, and ATF-6β (Figure 5E) that is associated with ER stress, thus, TET induced cell apoptosis may be through ER stress. Cells were pretreated with NAC and then treated with TET and the results show decreased GRP78, Caspase-12, ATF-6α, calpain 1 protein expressions that are associated with ER stress (Figure 5F). Catalase, GST, and SOD provide major antioxidant defenses against ROS. Furthermore, exposures of NPC-TW 076 cells to TET treatment caused decreased GST expression after 12 h and increased SOD (Cu/Zn) expression from 9 to 12 h (Figure 5G). As shown in Figure 5H, TET induced ER stress because we found that the expression of CHOP protein increase after 12 h treatment course and the expression of eIFG2α decrease after 6 to 48 h treatment. Other apoptotic proteins also revealed that TET induced apoptosis because of the increase of cleaved PARP protein from 6 to 48 h TET treatment and the increase of caspase 3 at 12 h treatment course.

### 2.6. TET Altered the Translocation of Apoptotic Associated Proteins in NPC-TW 076 Cells

**Figure 6:**
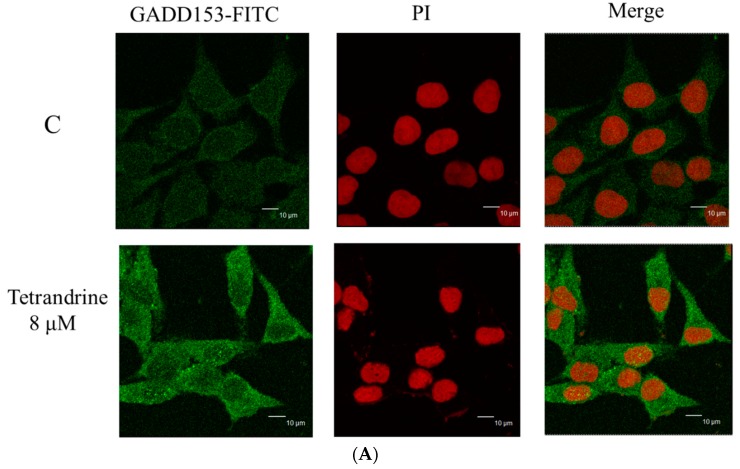

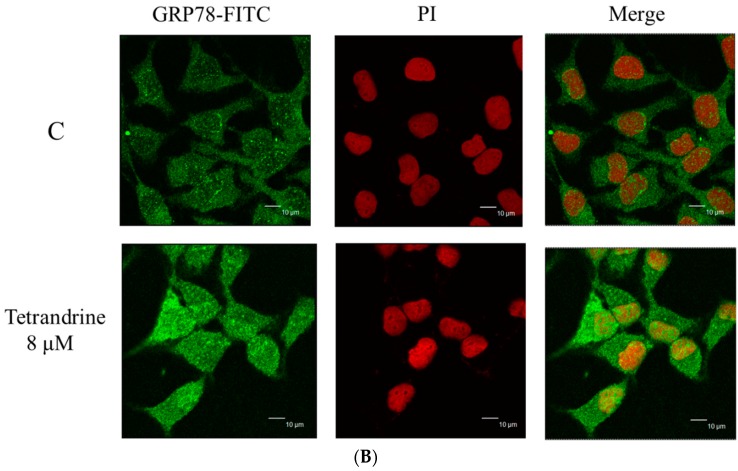
TET affects the translocation of apoptotic associated proteins in NPC-TW 076 cells. Cells were treated with 8 μM of TET for 24 h and cells were stained by anti- GADD153 (**A**) and GRP78 (**B**) and were stained with secondary antibody (FITC-conjugated goat anti-mouse IgG (green fluorescence) and PI (nuclear staining; red fluorescence) and followed by being examined and photographed by a Leica TCS SP2 confocal laser microscopic systems as described in Materials and Methods.

For further investigating the altering translocation of apoptosis associated protein such as GADD153 and GRP78 in NPC-TW 076 cells induced by TET, confocal laser microscopy was performed. Cells were treated with or without 8 μM of TET for 24 h, followed by staining with anti-GADD153 and GRP78 and then were photographed by confocal laser microscopic systems and the results are shown in Figure 6. These results indicated that TET treatment can increase the nuclear translocations of GADD153 (Figure 6A) and GRP78 (Figure 6B). Both proteins were translocated into nuclei when compare to untreated group.

## 3. Discussion

More than 90% of NPCs are undifferentiated carcinomas, thus, they show relative sensitivity to chemotherapy. These therapies have shown relatively high efficacy, however, and the survival of patients with late stage of NPC has not significantly improved [29,30,31]. Therefore, the identification of novel drugs from natural products is one of the strategies for improving the treatment options of patients with advanced NPC. Currently, although abundant evidence has shown that TET induces cell death in many human cancer cell lines, little is known about its effect on nasopharyngeal carcinoma cells. TET displays multiple anti-tumor activities including the inhibition of proliferation, angiogenesis, migration, and invasion, the induction of apoptosis, the reversal of multidrug resistance, and sensitization of radiation effects [28]. TET induces apoptosis in nasopharyngeal carcinoma CNE cells by down-regulating Bcl-2 mRNA and up-regulating Bax mRNA expression. TET also sensitizes CNE cells to irradiation-induced cytotoxicity and might abrogate radiation-induced G_2_ phase arrest [32]. However, there is no report to show the effects of TET on human nasopharyngeal carcinoma NPC-TW 076 cells. Herein, we investigate the cytotoxic effects of TET and associated possible signal pathways on human nasopharyngeal carcinoma NPC-TW 076 cells in vitro. Results indicated that TET reduced cell number via decreased cell viability and cell morphological changes (Figure 1A,C), induced G_0_/G_1_ phase arrest and sub-G_1_ phase (apoptotic cell death) (Figure 2A,B), and induced nuclear chromatin condensation (Figure 3A,B). Furthermore, flow cytometric assays showed that TET increased ROS and Ca^2+^ levels but did not significantly affect the levels of *ΔΨm* (Figure 4). Western blotting indicated that TET significantly increased the expression of calpain 1, calpain 2, caspase-12, IRE-1α, IRE-1β (Figure 5D), GADD153, GRP78, ATF-6α, and ATF-6β (Figure 5E) that is associated with ER stress. Thus, TET induced cell apoptosis may be through ER stress.

The anticancer properties of drugs sometimes come from the ability to induce cell cycle arrest, thus we determined the effects of TET on the cell cycle arrest. Flow cytometric analysis exhibited an increased accumulation of cells in the G_0_/G_1_ phase accompany with sub-G_1_ phase present in TET-treated group when compared to control group (Figure 3A,B). Further, we also used DAPI staining to show TET induces chromatin condensation which is also a characteristic of cell apoptosis. The mechanism of TET-mediated regulation of apoptotic and cell cycle-related genes require further investigation.

In order to further investigate the possible apoptotic signaling pathways induced by TET in NPC-TW 076 cells, cells were treated with TET and the production of ROS and Ca^2+^ were measured. We found that TET significantly increased the production of both ROS and Ca^2+^ and those effects are time-dependent. The balance between the accumulation of ROS and the activity of antioxidant system can partially regulate cell cycle progression, thus, drugs with the ability to interfere this balance can control the progression of cell proliferation [31]. The breakdown in oxidative phosphorylation and subsequently a decrease in ATP and loss of *Ψm* has been recognized to be one of the earliest events in apoptosis [12]. Reactive oxygen species (ROS) (by-products of normal cellular oxidative processes) act by regulating the processes involved in the initiation of apoptotic signaling. It has been shown that an increase in ROS generation in the mitochondria can induce cytochrome c release from mitochondria to the cytosol to mediate the apoptotic process [33]. The next experiment was used NAC (a ROS scavenger) to pretreat NPC-TW 076 cells before the TET treatment. We found that the NAC pretreatment can reduce the ROS production and had higher cell viability than TET treatment alone (Figure 4B,C). The overproduction of ROS can cause severe cellular damage and initiate the cancer development [34]. 

The endoplasmic reticulum (ER) is also a Ca^2+^ storage compartment of cells, and when suffering from ER stress, Ca^2+^ ions will be released into the cytoplasm followed by activation of numerous kinases and proteases [35]. ER stress has been recognized to be triggered by the accumulation of misfolded and/or unfolded proteins in the ER lumen and it leads to disruption of ER homeostasis. ER stress has been reported to be involved in specific transcriptional and translational responses that are largely controlled by three ER resident sensor proteins: inositol-requiring enzyme 1 (IRE1), ATF6, and PRKR-like endoplasmic reticulum kinase (PERK) [36,37,38]. Furthermore, ER stress has also been recognized to be a cellular response to anticancer treatment [39,40]. Our western blotting results indicated that TET significantly increased the expression of activating transcription factor 6α (ATF-6α), activating transcription factor 6β (ATF-6β), glucose-regulated protein 78 kDa (GRP78), GADD153 (Figure 5E), calpain 1 and 2, and caspase-12 (Figure 5D) that were documented to be the hall markers of ER stress. GRP78 and GADD153 proteins were increased in NPC-TW 076 cells after exposed to TET (Figure 5E). In addition to being a sensor of ER stress, GRP78 has been shown to serve as a gatekeeper to the activation of the ER stress transducers [41,42]. GADD153 is crucial for switching to pro-apoptotic signaling [43]. We also used confocal laser microscopy to confirm that TET promoted the translocation of GADD153 and GRP78 from cytoplasm to nuclei (Figure 6A,B). Therefore, we suggest that TET induces apoptotic cell death in NPC-TW 076 cells through the ER stress pathway. Our results also are consistent with the mechanism of the TET bromide derivative that induced ER stress and apoptosis on human non-small cell lung cancer cells [44].

**Figure 7:**
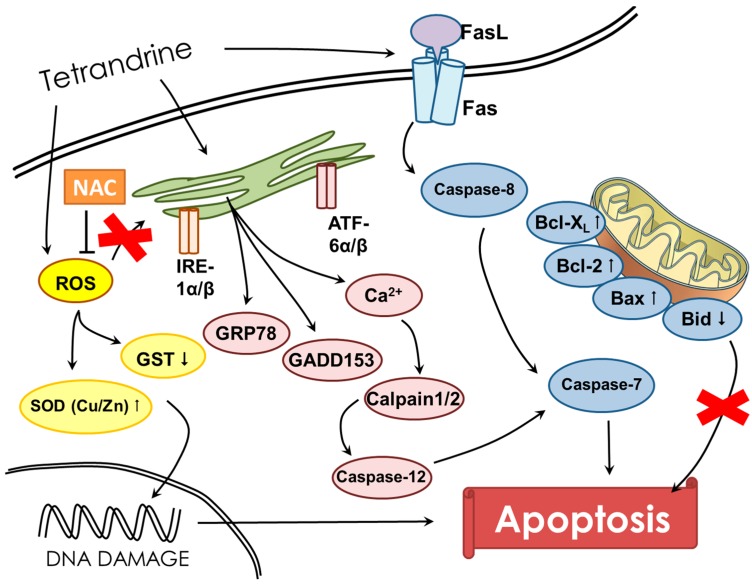
The possible signaling pathways for tetrandrine-induced apoptosis in NPC-TW 076 human nasopharyngeal carcinoma cells.

In conclusion, our results provide strong evidence to support the role of ER stress in mediating the apoptotic effect of TET on NPC-TW 076 cells in vitro. Treatment with TET induces expression of a number of signature ER stress markers. Overall, the possible pathways for TET induced cell apoptosis in NPC-TW 076 cells are summarized in Figure 7. These findings indicate the therapeutic potential of TET in treating human nasopharyngeal carcinoma in the future.

## 4. Experimental Section

### 4.1. Chemicals and Reagents

Tetrandrine (TET) of 99% purity, 4′,6-diamidino-2-phenylindole (DAPI), *N*-acetyl-l-cysteine (NAC), 4-phenylbutyrate (4PBA), dimethyl sulfoxide (DMSO), propidium iodide (PI) and Trypsin-EDTA were obtained from Sigma Chemical Co. (St. Louis, MO, USA). RPMI1640 medium, fetal bovine serum (FBS), l-glutamine and penicillin-streptomycin were purchased from GIBCO^®^/Invitrogen Life Technologies (Carlsbad, CA, USA). Primary antibody against caspase-7, -8 and -12, calpain 1, calpain 2, Fas, Fas-L, AIF, Endo G, XIAP, GADD153, GRP78, Catalase and GST were purchased from Santa Cruz Biotechnology, Inc. (Dallas, TX, USA) and cytochrome c, Bid, Bax, Bcl-2, Bcl-xL, IRE-1α, IRE-1β, ATF-6α, ATF-6β, SOD and peroxidase conjugated secondary antibodies were purchased from Cell Signaling Technology, Inc. (Beverly, MA, USA). TET was dissolved in DMSO. Cell culture grade DMSO as used for vehicle at 0.1%.

### 4.2. Cell Culture

The human nasopharyngeal carcinoma NPC-TW 076 cells line was purchased from the Food Industry Research and Development Institute (Hsinchu, Taiwan). NPC-TW 076 cells were grown in RPMI1640 medium supplemented with 10% heat inactivated fetal bovine serum (FBS) and antibiotics (100 unit/mL penicillin, 100 μg/mL streptomycin, and 2 mM glutamine). The cell lines were cultured in an incubator at 37 °C in a humidified atmosphere containing 5% carbon dioxide (CO_2_) [45]. 

### 4.3. Cell Viability and Morphology Examinations

Cell viability was measured using a flow cytometric assay. NPC-TW 076 cells were maintained at a concentration of 1 × 10^5^ cells/well in 12-well plates with RPMI1640 medium for 24 h. Cells were treated with TET at a concentration range of 0–9 μM for 48 h. After incubation, for total cell viability, cells were collected, counted and stained with PI (5 μg/mL) and then were measured by FACSCalibur flow cytometry (BD Biosciences, San Jose, CA, USA) and cells treated with TET at a concentration range of 0–10 μM for 48 h were examined for morphological changes and photographed under phase contrast microscopy at 200× as previously described [46]. 

### 4.4. Chromatin Condensation Stained with DAPI 

NPC-TW 076 cells (1 × 10^5^ cells/well) were placed in 12-well plates and cells were treated with TET at final concentrations (0, 4, 6, 8 and 10 μM) for 48 h. Cells were fixed in 3% paraformaldehyde in PBS for 20 min at room temperature, followed by washing with PBS. Cells were then stained with DAPI solution (2 μg/mL) at room temperature in the dark. The chromatin condensation (nuclear morphology) was examined and photographed using a fluorescence microscope as described previously [47].

### 4.5. Cell Cycle and Sub-G_1_ Phase (Apoptosis) Assays 

The percentage of cell cycle distribution and sub-G_1_ hypoploid cells were measured by flow cytometry as previously described [44]. Briefly, NPC-TW 076 cells (1 × 10^5^ cells/well) were maintained in 12-well plates and cells were treated with TET at final concentrations (0, 4, 6, 8 and 10 μM) for 48 h. Cells were collected, washed, fixed in 70% ethanol for 30 min, and incubated with a solution containing 50 mg/mL PI and 50 μg/mL RNase A in the dark at 37 °C. And then cells were analyzed for cell cycle distribution and sub-G_1_ phase by using flow cytometry and CellQuest and Mod Fit computer programs (BD Biosciences Clontech, Palo Alto, CA, USA) as described previously [47].

### 4.6. Measurements of Reactive Oxygen Species (ROS), Intracellular Ca^2+^ and Mitochondrial Membrane Potential (Ψm)

The levels of ROS, Ca^2+^ and *Ψm* in NPC-TW 076 cells after being exposed to TET were measured by flow cytometric assay. NPC-TW 076 cells (1 × 10^5^ cells/well) in 12-well plates and/or cells were pretreated with NAC or 4PBA for 3 h followed by treated with TET (8 μM) for various time periods. Cells were isolated to re-suspend with 500 μL of DCFH-DA (10 μM), 500 μL of DiOC6 (4 μmol/L) and 500 μL of Fluo-3/AM (2.5 μg/mL) for measuring ROS, ΔΨm and Ca^2+^ levels, respectively or were assayed for total viable cells by using flow cytometry as described previously [48]. 

### 4.7. Western Blotting Analysis 

NPC-TW 076 cells (1 × 10^6^ cells/well) were seeded in 10 cm dishes for 24 h and then were treated with TET (8 μM) for 0, 6, 12, 24 and 48 h. Cells were collected and lysed in lysis buffer (100 mM Tris-Cl, pH 6.8, 4% (*m*/*v*) SDS, 20% (*v*/*v*) glycerol, 200 mM β-mercaptoethanol, 1 mM phenylmethylsulfonyl fluoride, and 1 g/mL aprotinin). The total protein was measured by a protein assay kit (Bio-Rad, Hercules, CA, USA) as described previously. Then protein samples were separated with 12% (*v*/*v*) sodium dodeyl sulfate polyacrylamide gel electrophoresis (SDS-PAGE) and transferred onto the PVDF membranes (Millipore, Billerica, MA, USA). Immune complexes were formed by incubation of proteins with primary antibodies overnight at 4 °C followed by a peroxidase-conjugated anti-mouse IgG (Cell Signaling Technology). Immunoreactive protein bands were detected using the enhanced chemiluminescence detection system (GE Healthcare Bio-Sciences, Pittsburgh, PA, USA) [48,49]. Protein expression was quantified by densitometry using Image J.

### 4.8. Confocal Laser Scanning Microscopy Assay 

Confocal laser microscopy (Leica Inc., Heidelberg, Germany) was used to examine protein translocations as described previously. Briefly, NPC-TW 076 cells (5 × 10^4^ cells/well) in 4-well chamber slides were treated with TET (0 and 8 μM) for 24 h. And 4% formaldehyde in PBS was used to fix for 15 min followed by incubation with 0.3% Triton-X 100 in PBS for 1 h to make the cells permeable and then 2% BSA were used for blocking non-specific binding sites. Cells were incubated with primary antibodies such as anti-GADD153 and -GRP78 for overnight and cells were stained with secondary antibody (FITC-conjugated goat anti-mouse IgG) and PI (red fluorescence) staining for nuclear examinations. Slides were mounted, examined and photographed under a TCS SP2 Confocal Spectral Microscope (Leica Inc.) as described previously [50].

### 4.9. Statistical Analysis

Results were presented as mean ± standard deviation (SD) from triplicate experiments. Statistical analysis of all data was performed by Student’s t test. All comparisons are made relative to untreated controls and significance of difference is indicated as * *p* < 0.05, ** *p* < 0.01, and *** *p* < 0.001.
